# The NRF2-KEAP1 Pathway Is an Early Responsive Gene Network in Arsenic Exposed Lymphoblastoid Cells

**DOI:** 10.1371/journal.pone.0088069

**Published:** 2014-02-07

**Authors:** Emilio J. Córdova, Angélica Martínez-Hernández, Laura Uribe-Figueroa, Federico Centeno, Mirna Morales-Marín, Harsha Koneru, Matthew A. Coleman, Lorena Orozco

**Affiliations:** 1 Immunogenomics and Metabolics Disease Laboratory, Instituto Nacional de Medicina Genómica, SS, México City, México; 2 Posgrado en Ciencias Genómicas, Universidad Autónoma de la Ciudad México, México City, México; 3 Lawrence Livermore National Laboratory, Livermore, California, United States of America; 4 Department of Radiation Oncology, Davis Medical Center, University of California Davis, Sacramento, California, United States of America; North Carolina State University, United States of America

## Abstract

Inorganic arsenic (iAs), a major environmental contaminant, has risen as an important health problem worldwide. More detailed identification of the molecular mechanisms associated with iAs exposure would help to establish better strategies for prevention and treatment. Although chronic iAs exposures have been previously studied there is little to no information regarding the early events of exposure to iAs. To better characterize the early mechanisms of iAs exposure we conducted gene expression studies using sublethal doses of iAs at two different time-points. The major transcripts differentially regulated at 2 hrs of iAs exposure included antioxidants, detoxificants and chaperones. Moreover, after 12 hrs of exposure many of the down-regulated genes were associated with DNA replication and S phase cell cycle progression. Interestingly, the most affected biological pathway by both 2 or 12 hrs of iAs exposure were the Nrf2-Keap1 pathway, represented by the highly up-regulated *HMOX1* transcript, which is transcriptionally regulated by the transcription factor Nrf2. Additional Nrf2 targets included *SQSTM1* and *ABCB6*, which were not previously associated with acute iAs exposure. Signalling pathways such as interferon, B cell receptor and AhR route were also responsive to acute iAs exposure. Since *HMOX1* expression increased early (20 min) and was responsive to low iAs concentrations (0.1 µM), this gene could be a suitable early biomarker for iAs exposure. In addition, the novel Nrf2 targets *SQSTM1* and *ABCB6* could play an important and previously unrecognized role in cellular protection against iAs.

## Introduction

Inorganic arsenic (iAs) is one of the most important natural pollutants worldwide [1]. iAs accumulates in groundwater from both industrial waste disposal sites and natural sources. Thus, human exposure occurs mainly through contaminated drinking water [2]. Epidemiological studies have highlighted a close correlation between chronic exposure to iAs and increased incidences of various illnesses such as diabetes, cardiovascular diseases and cancer [3], [4], [5]. Several reports have showed that iAs has a potent immunosuppressive effect, which could participate in the pathological properties associated with iAs exposure [6], [7], [8]. Among the toxic effects of iAs, the generation of reactive oxygen species (ROS) and the direct interaction with proteins and epigenetic alterations have been previously proposed [9], [10], [11].

Several reports suggest that Nrf2 (Nuclear factor (erythroid-derived 2)-like 2)-Keap1 (kelch-like ECH-associated protein 1) signalling pathway is one of the most important cellular mechanisms against iAs exposure. This pathway, regulated by the transcription factor Nrf2, has been involved in cellular protection against a myriad of toxic products including metalloids, chemical carcinogens and radiation [12], [13], [14]. The cytoprotective function of Nrf2 is based on the regulated expression of a battery of genes associated with oxidative stress, chronic inflammation and cellular detoxification [15]. Activation of Nrf2 after chronic exposure to iAs has been demonstrated in different cell types including hepatocytes, keratinocytes and osteoblasts [16], [17], [18]. However, the participation of signalling pathways other than Nrf2-Keap1 in the cellular response against iAs, particularly at acute exposure remains unclear.

A better knowledge about signalling pathways involved in the cellular response against iAs at environmentally relevant levels could help in the identification of biomarkers of early exposure for iAs and the improvement of strategies for reducing the cellular toxicity related to this metalloid. Microarray analysis is a useful tool to determine whole gene expression induced by both external and internal stimuli, in order to identify important pathways. In-spite of various reports highlighting the immunosuppressive action of iAs [6], [7], [8], most of the previously performed microarray analysis on iAs exposure were carried out in epithelial derived cell lines [19], [20], [21]. Thus, this study focuses on identification and characterization of the early molecular responses against sublethal dose of iAs using human derived lymphoblastoid cells. We found that Nrf2-Keap1 pathway is not the only route responsive to acute doses of iAs in lymphoblastoid cells. In addition, this study indicates that the *HMOX1* (heme oxygenase (decycling) 1) transcript could function as both an acute and chronic biomarker of arsenic exposure.

## Methods

### 2.1 Cell Culture

We used human lymphoblastoid primary cultures and two human lymphoblastoid cell lines (CL-1 and CL-45), independently immortalized with Epstein-Barr virus (catalog No. VR-1492; ATCC, Manassas, VA). All cell cultures were propagated in RPMI media supplemented with 10% fetal bovine serum (FBS), L-glutammine 2 mM and antiobiotics (10 000 U/ml penicillin and 10 mg/ml streptomycin) (Invitrogen Calrsbad, CA) at 37°C in an atmosphere of 5% CO_2_. In the case of primary cultures, 1% phytohemaglutinine were added. Cell cultures (1×10^6^) were treated with 5 µM sodium arsenite (Sigma, St. Louis, MO) for 2 and 12 hrs while control cultures were incubated with PBS. Nine biological replicates for each condition were used for microarray analysis. This study was approved by the respective ethics and human research committees of the National Institute of Genomic Medicine at Mexico and all participants signed an informed consent agreement.

### 2.2 Microarray-based Gene Expression Analysis

After iAs treatment, total RNA from each biological replicate was isolated with Trizol following manufacturers instructions (Invitrogen, Carslaban CA). RNA was quantified with a Nanodrop spectrophotometer (Nanodrop Technologies, Wilmington, DE) and quality control was assessed by RNA 6000 Nano Chips, and analyzed on an Agilent 2100 Bioanalyzer (Agilent Technologies, Santa Clara, CA). From the 9 biological replicates performed on each condition, we generated 3 pools at a concentration of 300 ng/µL, where each pool was processed to a single microarray platform. Target cDNA was prepared according to the Whole-Transcript (WT) Sense Target Labeling Protocol and hybridized to the Human Gene 1.0 ST microarray (Affymetrix, Santa Clara CA). The GeneChip Scanner 3000 7G collected fluorescent signals, and the Expression Console software subsequently amassed intensity and quality data on the scanned arrays (Affymetrix, Santa Clara, CA).

### 2.3 Microarray Data Analysis

Signal intensity from the 9 arrays in.CEL format was analyzed using Genomatix Software Suite version 6.4 (Genomatix, Germany). To identify differentially expressed genes, one-way ANOVA using a Benjamini and Hochberg false discovery rate (P<0.05) was performed, followed by a Tukey–Kramer post hoc test (P<0.05). Genes with *P*<0.05 and a differential fold change >1.5 in either direction were selected for functional characterization. Our experiment was performed in compliance with the Minimum Information About a Microarray Experiment (MIAME) checklist and followed standardization guidelines for microarray experiments (GEO accession number GSE51454) [22]. We selected differentially expressed genes with a false discovery rate (FDR) <5% from a delta value of 0.7 after 1,000 permutations.

### 2.4 Reverse Transcription-real-time Quantitative PCR (RT-qPCR) Analysis

Validation of specific transcripts in CL-1 and CL-45 cell lines was carried out by reverse transcription-real-time polymerase chain reaction (RT-qPCR) using SYBR green as detection method with transcript specific primers. After cDNA preparation with MultiScribe Reverse Transcriptase (Applied Biosystem, Foster, CA), a 1∶5 dilution was used as a template for PCR amplification. *GAPDH* (Glyceraldehyde 3-phosphate dehydrogenase) was used as an endogenous control and all PCR reactions were performed in triplicate. Fold-change of transcripts in samples exposed to 5 µM sodium arsenite for 2 or 12 hrs relative to transcripts levels in untreated samples was determined by the average of the Δ∶CT method [23].

### 2.5 Functional Annotation and Canonical Pathways

Genes differentially expressed after iAs exposure were functionally annotated using the Database for Annotation Visualization and Integrated Discovery (DAVID) Gene Ontology (GO) search engine (http://www.geneontology.org/GO.tools.microarray.shtml). In addition, we determine gene networks and canonical pathways significantly affected by iAs exposure using the curated database Ingenuity Pathways Analysis (IPA).

### 2.6 Analysis of Nrf2-regulated Genes in Response to iAs Exposure

The induction of *HMOX1*, *SQSTM1* (sequestosome 1), *GSTM* (glutathione S-transferase M), *GCLM* (glutamate-cysteine ligase, modifier subunit), *FTL* (ferritin light-chain), *GSR* (glutathione reductase), *MRP2* (multidrug resistance protein 2) and *NQO1* (NAD(P)H dehydrogenase, quinone 1) genes after iAs treatment was evaluated for time-response (0, 4, 8, 12 and 24 hrs) in CL-1 cell line. In addition, *HMOX1* and *NQO1* were also analyzed in dose-response (0.1, 0.5, 1.0, 2.5 and 5.0 µM for 12 hrs) and a shorter time-response (20, 40, 60, 80, 100 and 120 min). The expression levels of both *HMOX1* and *NQO1* were also determined in primary cultures treated with 5.0 µM of sodium arsenite for 12 hrs.

### 2.7 Western Blot Assays

Lymphoblastoid cells were pelleted and washed in PBS. Cells were then lysed in 200 µL of M-Per (Pierce-Thermo Fisher) following the manufacturers published protocol. Samples were quantified using a Qubit spectrophotometer (Life Technologies). A 25 µg sample was taken and re-suspended in NuPage loading buffer and run on a 4–12% NuPAGE gel (Life Technologies). The running buffer was 1X MES-SDS (Life Technologies Corporation, Carlsbad, California). Western blotting and Imaging analysis used LI-COR published protocols and an Odyssey Imager. All experiments were conducted in triplicated and blotted in duplicate. The antibodies against Nrf2 (sc-722), HMOX1 (sc-10789) and NQO1 (sc-16464) were purchased from Santa Cruz Biotechnology and used at 1/100 dilutions. The beta Actin control (A0760-40) was purchased from U.S. Biological (Swampscott, MA.), and used at a dilution of 1/1000. Secondary antibodies (IRDye 800CW and IRDye 680LT) were used at a dilution of 1/5000 and were purchased from LI-COR.

### 2.8 Statistical Analysis

All independent gene expression and RT-qPCR assays were performed in triplicate. Data are expressed as means ±SD. Significant differences were evaluated using the Student *t*-test or the multirange Dunett’s *t*-test when multiple comparisons were studied.

## Results

**Figure 1:**
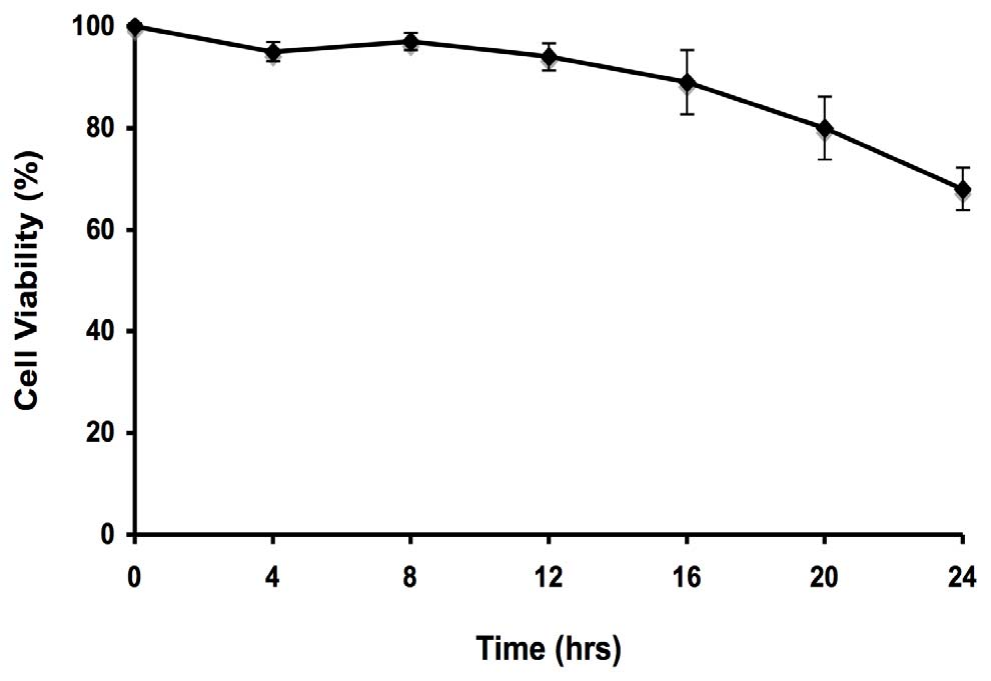
Transcripts altered by iAs exposure. Volcano plots of differential transcripts up- (black dots) and down-regulated (grey dots) after (A) 2 and (B) 12 hrs of exposure to 5 µM of iAs.

To explore the cellular mechanisms associated with iAs exposure, CL-1 human non-tumoral lymphoblastoid cells were treated with a sublethal dose of iAs (as sodium arsenite, 5 µM) for either 2 or 12 hrs. The cytotoxic effects of iAs were evaluated by trypan blue exclusion assay (Figure S1). A comparison between unexposed and exposed samples using significance analysis with the Genomatix software suit identified 299 differential transcripts (Figure 1). A higher number of genes showed altered expression with the longer time of exposure. We found 81 genes with altered expression at 2 hrs (33 down-regulated and 48 up-regulated) and 218 altered genes at 12 hrs (153 down-regulated and 65 up-regulated). The complete list of differential gene expression is shown in Table S1 and Table S2 (GEO accession number GSE51454).

**Figure 2:**
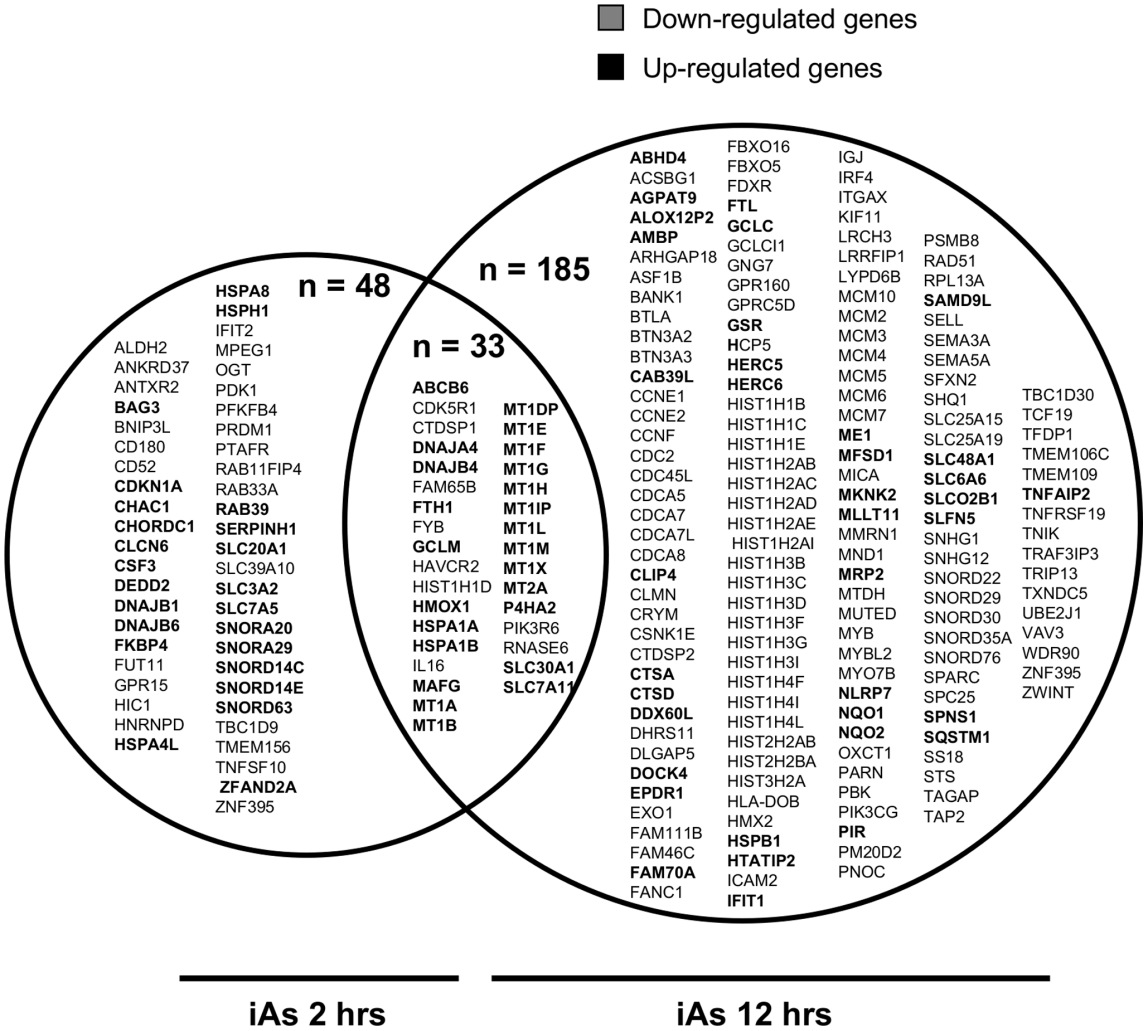
Comparison of altered genes at different times after iAs exposure. Venn diagram depicting genes affected exclusively at 2(left circle), 12 hrs of exposure to 5 µM of iAs (right circle) and both.

From the total of differential transcripts altered by 2 or 12 hrs of exposure, 33 showed a difference in their expression at both doses, 48 were exclusively affected at 2 hrs and 185 only after 12 hrs of exposure (Figure 2). Up-regulated genes observed at 2 hrs of exposure consisted of various members of the small nucleolar RNA family (*SNORA20* and *SNORD63*), intracellular signalling (*CSF3* and *SERPINH1*), stress response (*DNAJB1*) and solute carriers (*SLC3A2)*. In the case of the down-regulated transcripts at 2 hrs, we observed several genes involved in cellular metabolism (*ALDH2*, *FUT11* and *PFKFB4*) and immune regulation (*CD180*, *CD52* and *IFIT2*). At 12 hrs, we also found up-regulated antioxidant (*NQO1* and *GCLC*) and solute carrier genes (*SLC6A6* and *SLCO2B1*); while genes enrolled in immune regulation (*CDC45L*, *HLA*-*DOB* and *IRF4*), histone metabolism (*HIST1H1B*, *HIST1H2AB* and *HIST1H3B*) and cell cycle regulation (*CCNE1*, *CDC2* and *MCM7*) were down-regulated.

The most prominent genes with increased expression at both times of exposure were the following: antioxidants genes such as *HMOX1*, *GCLM* and several members from the metallothionein family (*MT1A*, *MT1B*, *MT1D*, *MT1E*, *MT1F*, *MT1G*, *MT1H, MT1X*, and *MT2A*); stress response genes including *DNAJB4* and *HSPA1B*; as well as the solute carrier encoding genes *ABCB6*, *SLC30A1* and *SLC7A11*. Common genes with decreased expression between the two exposure periods were involved in immune regulation (*IL16, FYB* and *HACVR2*), DNA packaging (*HIST1H1D*), cell signalling (*PIK3R6, CDK5R1*) and RNA metabolism (*CTDSP1* and *RNASE6*).

**Figure 3:**
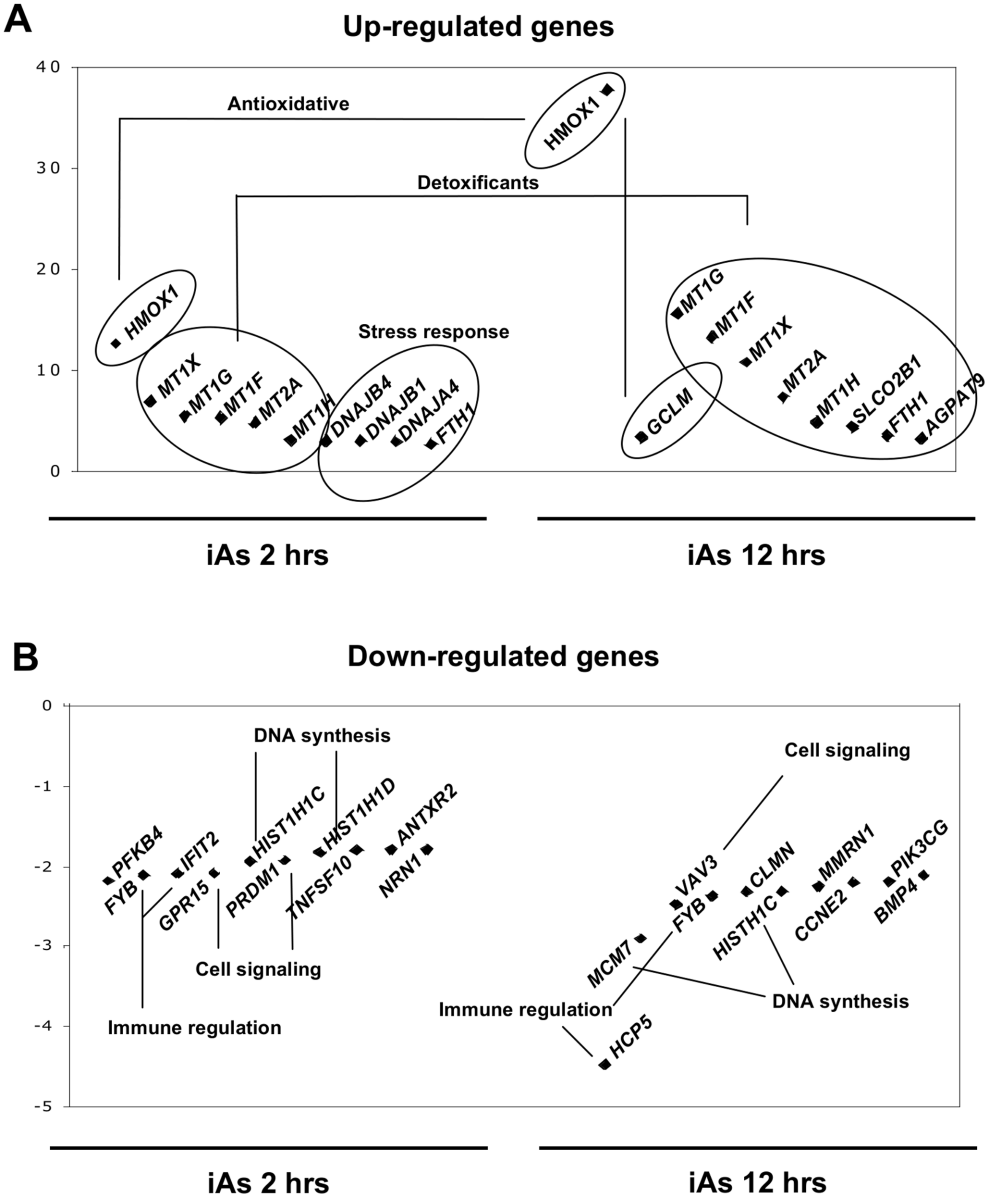
Major genes altered by iAs exposure. Cellular functions performed by the proteins encoded for the ten most up-regulated (A) and down-regulated (B) genes after 2 and 12 hrs of exposure to 5 µM of iAs.

When we compared the most up-regulated genes at 2 hrs with those from 12 hrs (Figure 3A), we observed that expression of *HMOX1, MT1X*, *MT1F, MT1G* and *MT2A* was significantly higher at 12 than 2 hrs of exposure (p<5×10^−5^). Other antioxidants genes that showed significant increase of expression at 12 hrs compared to 2 hrs were *SLCO2B1* and *GCLM* (p<0.001), although they were not among the most up-regulated genes after the short exposure. On the other hand, genes with decreased expression at both exposure times showed no significant differences in expression after 12 hrs of iAs exposure compared to 2 hrs (Figure 3B).

**Figure 4:**
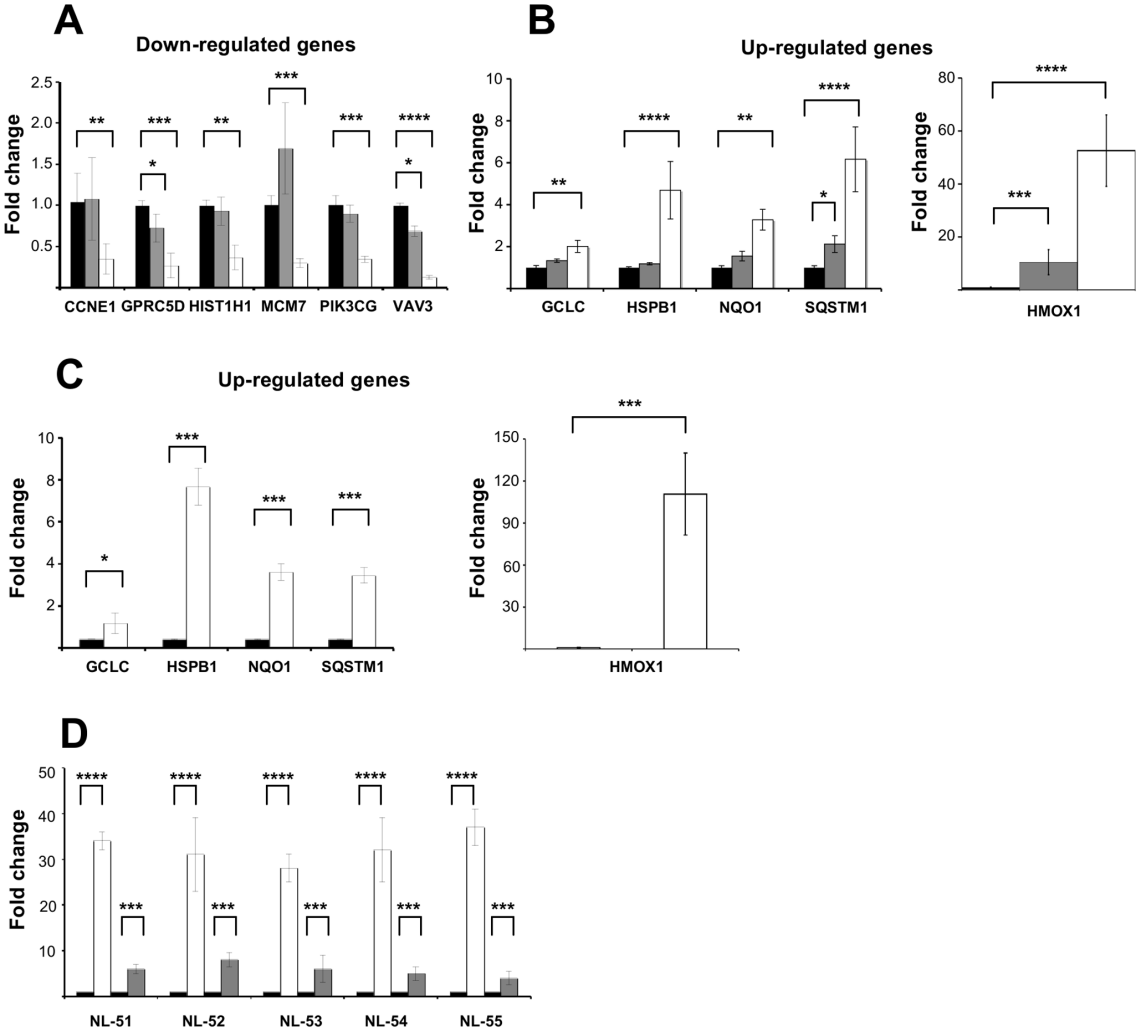
Validation of genes with altered expression after iAs exposure. Total RNA from CL-1 cell lines exposed to 5 µM of iAs for 2 and 12 h of exposure as indicated was used as template for RT-qPCR assays. Control: black bars, 2 hrs: grey bars and 12 hrs: white bars. A) Down-regulated genes, B) Up-regulated genes, C) Overexpressed genes in CL-45 cell lines treated with 5 µM of iAs for 12 hrs. Control: black bars, treated: white bars. D) Expression levels of *HMOX1* and *NQO1* genes in lymphoblastoid primary cultures with 5 µM of iAs for 12 hrs. Control: black bars, *HMOX1*: white bars and *NQO1*: grey bars. Data values are means ± SD and were normalized using *GADPH* as an endogenous gene. Fold change was related to untreated samples. Three independent cell cultures were used for each assay. **** *p*<0.001, *** *p*<0.005, ** *p*<0.01, * *p*<0.05.

Validation by RT-qPCR showed that the direction of the mRNA expression for all genes tested was similar in both the microarray and RT-qPCR assays (Figure 4A and 4B). However, the sensitivity of the RT-qPCR assays was higher than microarray-based measurements. For instance, the *GPRC5D* and *VAV3* genes showed significant decrease in expression at both 2 and 12 hrs by RT-qPCR, but only at 12 hrs by microarray (Figure 4A). Likewise, the *SQSTM1* gene showed a significant up-regulation at 2 hrs only by RT-qPCR assay (Figure 4B). These findings were reproduced when the expression of several up-regulated genes was determined by RT-qPCR in an additional lymphoblastoid cell line (CL-45) (Figure 4C). Moreover, to avoid a possible bias by the Epstein-Barr immortalization process of cell lines, expression levels of *NQO1* and *HMOX1* genes were tested in primary lymphoblastoid cultures exposed to iAs 5 µM for 12 hrs. The results showed a similar finding, and thus indicated that our observations were biologically relevant for our model system (Figure 4D).

**Figure 5:**
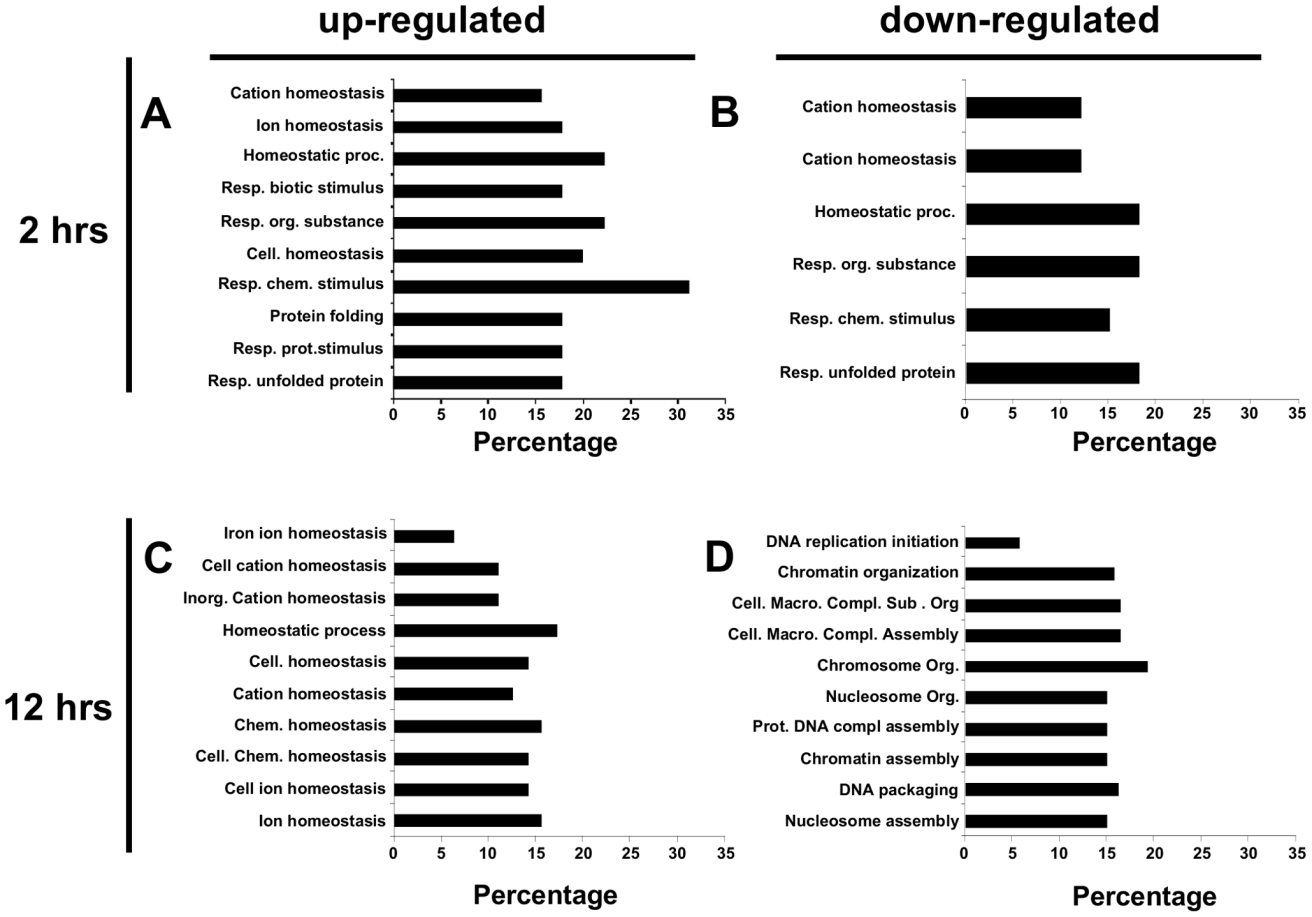
Biological processes altered by iAs exposure. Most significant Gene Ontology (GO) terms associated with gene expression up-regulation (A–C) and down-regulation (B–D) after 2 (A, B) and 12 hrs (C, D) of exposure to 5 µM of iAs. All selected categories showed a p<0.001.

Functional annotation clustering of iAs altered genes by DAVID showed enrichment for 59 GO categories at 2 hrs of exposure and 94 GO categories at 12 hrs. After selecting categories with a *p*<0.001, the up-regulated genes at 2 hrs of iAs exposure were classified in several biological processes involved in cellular protection such as “homeostatic process” (10 genes), “response to chemical stimulus” (14 genes) and “response to unfolded protein” (8 genes), among others (Figure 5A). At 12 hrs of exposure, the functional categories enriched with up-regulated genes also included mainly homeostatic process (Figure 5C).

In the case of the down-regulated genes, 2 hrs of exposure yielded only 6 statistically enriched GO categories three related with cellular homeostasis and three with response to stress (Figure 5B). Interestingly, at 12 hrs most of the down-regulated biological processes were associated with DNA structure and replication, such as “chromatin organization” (22 genes), “nucleosome organization” (21 genes), “protein-DNA complex assembly” (21 genes), “chromatin assembly” (21 genes) and “DNA packaging” (23 genes) (Figure 5D).

**Figure 6:**
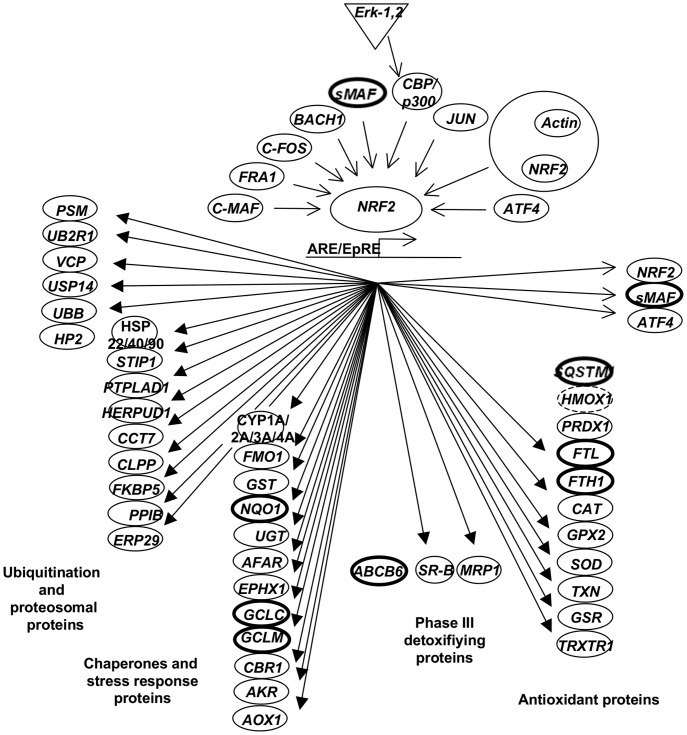
The Nrf2-Keap1 regulatory pathway and iAs responsive transcripts. Known and novel iAs responsive Nrf2 transcripts are shown. Empty circles depict unaffected target transcripts (not differentially expressed in lymphoblastoid cells). Bold and no continuous line circles indicate the level of increased gene expression as identified within this study. Bold line = 2–5 fold, no continuous line = >10. Bold letter depicted novel Nrf2-target genes identify by microarrays.

To further identify significant canonical pathways associated with iAs exposure we used the Ingenuity Pathway Analysis software suite (Table 1). We found that in both exposure periods used in this study, the Nrf2-mediated oxidative stress response was the canonical pathway with the most statistically significant level of enrichment; in response to iAs exposure, several target genes of Nrf2 showed an increased expression (Figure 6). Other canonical pathways affected at 2 hrs of exposure were the interferon signalling, B-cell receptor signalling, glucocorticoid receptor signalling and FC gamma receptor signalling pathways. On the other hand, the 12 hrs exposure altered the aryl-hydrocarbon receptor signalling (AhR), glutathione metabolism, glutamate metabolism and one carbon pool by folate.

**Table 1:** Canonical pathways associated with iAs exposure.

	0 *vs.* 2 hs		0 *vs.* 12 hs
Canonical pathway	*P* value	Canonical pathway	*P* value
NRF2-mediated Oxidative Stress response	1.36e-08	NRF2-mediated Oxidative Stress response	3.38e-08
Interferon signalling	2.32e-03	Aryl Hydrocarbon receptor signalling	4.88e-05
B cell receptor signalling	2.61e-03	Glutathione Metabolism	4.33e-03
Glucocorticoid receptor signalling	1.11e-02	Glutamate Metabolism	5.89e-03
Fc gamma receptor-mediated phagocytosisin macrophages and monocytes	1.28e-02	One carbon pool by folate	8.40e-03

**Figure 7:**
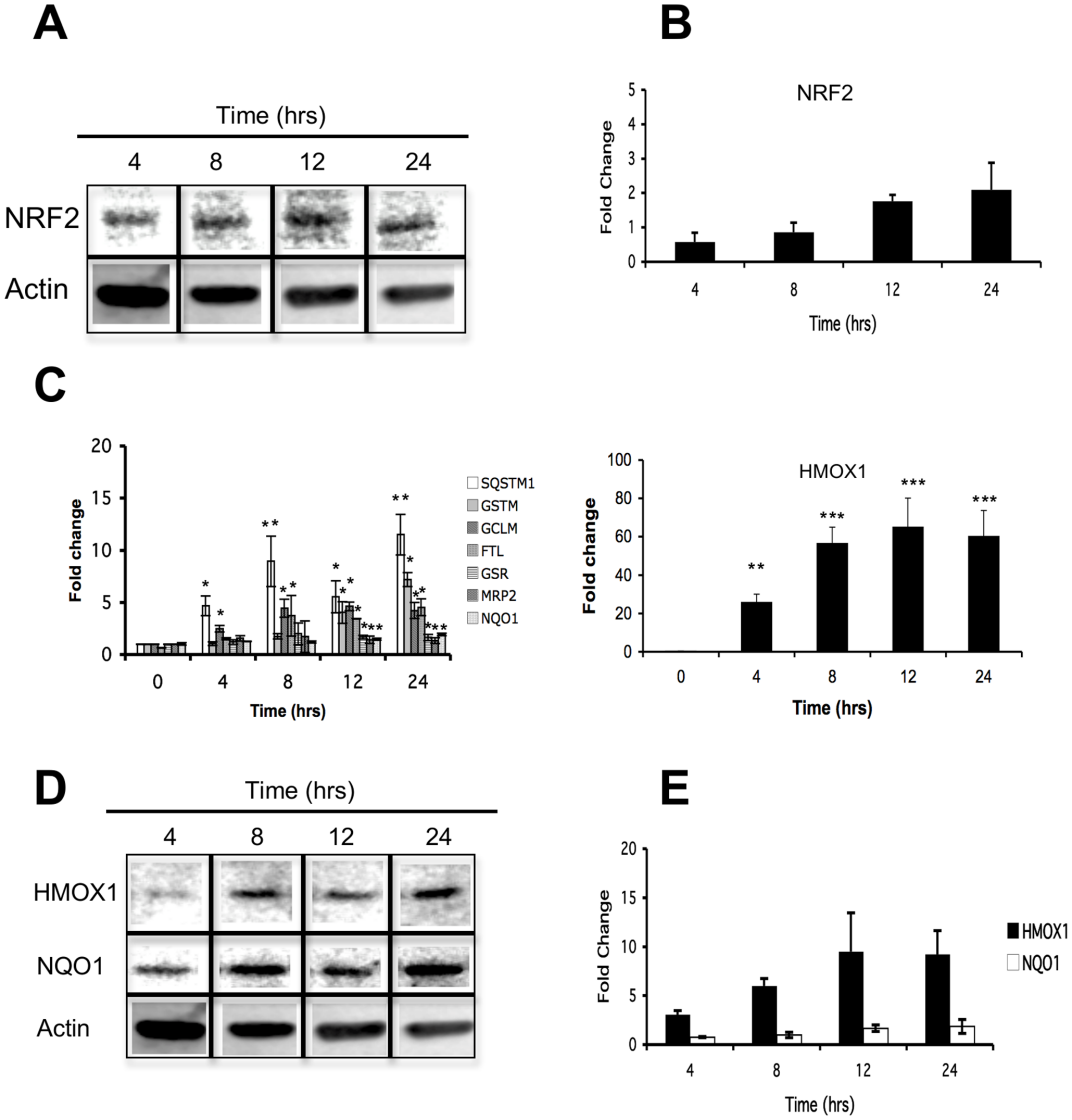
Response of Nrf2 and Nrf2-regulated genes to iAs exposure. Evaluation of Nrf2 protein and its respective target genes by Western blot and RT-qPCR in CL-1 cell line after exposure to 5 µM of iAs in a time-response curve. A) Nrf2 and beta-actin protein expression levels, 4–24 hours, were measured by Western blot as shown. B) Normalized levels of Nrf2 were compared to non-exposed samples and converted to fold-change and plotted to show an increase in Nrf2 expression in the presence of 5 µM of iAs. C) Validation of down stream Nrf2-regulated mRNA expression levels. D) HMOX1, NQO1 and beta-actin protein expression levels, 4–24 hours, were measured by Western blot. E) Normalized levels of HMOX1 and NQO1 were compared to non-exposed samples and converted to fold-change and plotted to show an increase in protein levels in the presence of 5 µM of iAs. Data values are means ± SD. Three independent cell cultures were used for each assay data point. ***p<0.005, **p<0.01, *p<0.05.

Western blots were used to profile induction of the Nrf2 transcription factor as well as the increased expression level of several genes regulated by Nrf2 over a 12 hrs period. We found a time-dependent fold increases in total protein levels of Nrf2 compared to untreated samples (Figure 7A and B). Accordingly, Nrf2-regulated expression levels of *HMOX1*, *SQSTM1*, *GSTM*, *GCLM* and *FTL* genes also showed a time-dependent increase, while the *GSR*, *MRP2* and *NQO1* transcripts showed a mild induction of their expression along the entire exposure time (Figure 7C). Corroborating the biological relevance of this transcriptional increase, we also observed an increase in the protein levels of HMOX1 and NQO1 after iAs exposure. The proteins HMOX1 and NQO1 expression profiles were very similar to their respective gene transcript expression patterns (Figure 7D and E).

**Figure 8:**
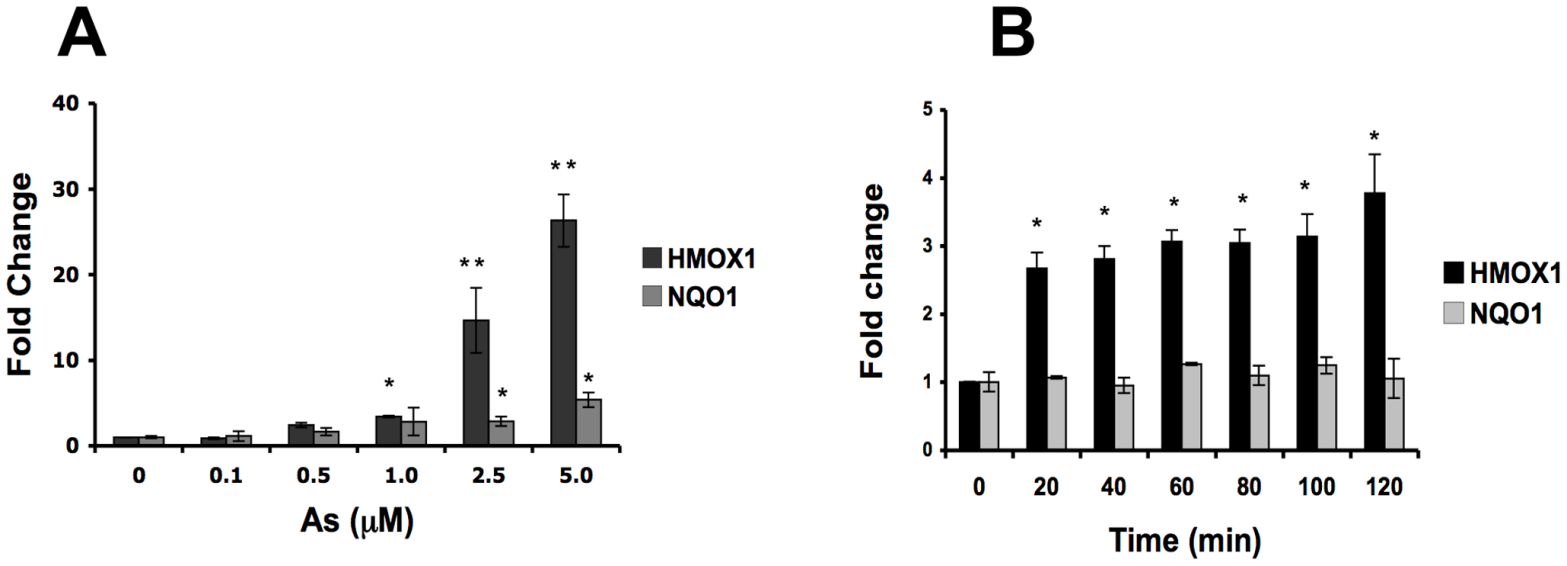
Response of Nrf2-regulated genes to iAs exposure. Evaluation of mRNA levels of the indicated Nrf2-target genes by RT-qPCR in CL-1 cell line after exposure to 5 µM of iAs in a time-response curve. A) *HMOX1* and *NQO1* expression after dose-response exposure at 12 hrs, B) *HMOX1* and *NQO1* time response at 5 µM of iAs. Data values are means ± SD and were normalized using *GADPH* as an endogenous gene and fold changes were calculated using the ΔΔCt method. Three independent cell cultures were used for each assay data point. **p<0.001, *p<0.01.

The mRNA levels of *HMOX1*, the highest responsive gene, rose in response to iAs in a dose-dependent manner at the 12 hr time-point and was responsive to concentrations as low as 0.1 µM and as high as 5 µM (Figure 8A). Moreover, *HMOX1* gene expression showed a time-dependent increase to 5 µM iAs since 20 min (Figure 8B). Besides, the induction of *HMOX1* expression was not diminished by the presence of iAs-associated cytotoxicity (Figure S1). Interestingly, *NQO1*, another NRF2 target gene that is known to be responsive to iAs, did not show the same type of expression patterns as *HMOX1,* suggesting that induction of *HMOX1* for iAs exposure may involve additional regulatory factors beyond Nrf2 signaling activation (Figure 7D–E and 8).

## Discussion

Despite the important participation of iAs in the pathogenesis of several human diseases, the early response molecular mechanisms involved in cellular protection against this metalloid are incomplete. In our experiments, iAs exposure produced an increase in different cytoprotective genes, such as members of the metallothionein family (e.g. *MT1A*, *MT1E* and *MT2A*) and the glutathione metabolism pathway (e.g. *GCLM* and *GCLC)*, as well as antioxidants (e.g. *HMOX1*, *NQO1* and *FTL*). A direct consequence of free radicals produced by iAs is the modification of different intracellular proteins. This iAs effect was evident in our study based on the expression of several associated chaperones (e.g. *DNAJB1* and *DNAJA4*) and heat shock genes (e.g. *HSPA8* and *HSPB1*), which can be induced by the increased presence of proteins with altered conformations [24], [25], [26]. The modification induced by iAs exposure to heat-shock proteins has been observed in acute iAs exposure in several other animal and cell culture studies as well as in chronically arsenic-exposed individuals, suggesting the importance of protein modification by iAs [27], [28], [29].

Our study highlights the down-regulation of genes implicated in DNA replication and immune system regulation after iAs exposure. The inhibition of DNA replication after iAs exposure has previously been documented in different cell types and may reflect the attempt of the cell to evade iAs-associated mutations by avoiding the replication of DNA damage [30], [31]. The immunosuppressive effect of iAs, previously described in individuals chronically exposed to iAs but not in acute exposures, has been proposed as a mechanism to promote immune escape and tolerance of tumors [32].

Since the Nrf2-Keap1 pathway is highly induced by iAs in different cell types [17], [18] and its pharmacological or genetic activation is able to reduce the toxic effects associated with this metalloid [33], [34], it has been proposed as the main cytoprotective mechanism against iAs. Our data analysis suggests that pathways other than Nrf2-Keap1, such as interferon, B cell receptor and AhR route are also responsive to acute iAs exposure. However, the route regulated by Nrf2, as previously reported, was also the most important early signalling pathway that was responsive to iAs based on our expression data in the lymphoblastoid cells. Interestingly, induction of several Nrf2 target transcripts were not previously observed in lymphocytes obtained from individuals chronically exposed to iAs through drinking-water [35], [36], [37]. This observation combined with our data, suggest that Nrf2-Keap1 pathway is an early mechanism against iAs exposure and that a process of cellular tolerance may turn off its activation after extended exposures to the metalloid in immune-related cells.

The Nrf2 regulated genes induced by iAs seems to partially depend on the cell type. For instances, the induction of *HMOX1*, *NQO1*, *GCLM* and *GCLC* transcripts by iAs has been previously reported in different cell types including monocytes, keratinocytes, kidney, endothelial and urothelial cells, among others [19], [21], [38], [39], [40]. On the other hand, the up-regulation of *FTL* and *FTH1* genes were only seen studies using monocytes and keratinocytes [39], [40], [41]. Our study is the first using microarrays to report the induction of *SQSTM1* and *ABCB6* genes, which are regulated by Nrf2, after iAs exposure. Similarly, other known Nrf2-regulated genes such as *ALDH2*, *GPX2*, *GSR*, *AKR* and *SLC* that have been previously reported to be induced by acute exposure to iAs in different cell types were not detected within our study [19], [20], [28]. This once again highlights the importance of the need to characterize both the early time points and acute *vs.* chronic exposures for identifying molecular signatures of response.

The *SQSTM1* and *ABCB6* genes may play an important role in cellular response to iAs. The p62/SQSTM1 protein (sequestosome 1) also known as A170 is an adaptor protein that mediates the degradation of ubiquitinated targets and is an important regulator of autophagic cell death [42]. The p62/SQSTM1 protein acts as a scaffold molecule in several signalling pathways including NF-kB and Nrf2 routes. Through the interaction with Keap1, p62/SQSTM1 protein induces the activation of Nrf2, which in turn stimulates expression of the *SQSTM1* gene establishing a positive feedback loop [43]. Exposure of osteoblast cells to sublethal doses of iAs induces the transcriptional activation of Nrf2 and the expression levels of p62/SQSTM2 [16]. The transporter protein ABCB6 (ATP-binding cassette transporter B 6) was originally identified in a screening for drug-resistant genes in the liver [44]. ABCB6 is capable of protecting cells against several sources of oxidative stress by regulating *de novo* biosynthesis of porphyrin [45], [46]. In a recent report, Chavan et al [47] showed that *ABCB6* expression was induced by arsenite exposure in an Nrf2-independent manner in human hepatocytes cells. Nevertheless, using a ChIP-seq approach *ABCB6* was identified as an Nrf2-target gene in human lymphoid cells treated with the chemiopreventor sulforaphane [48]. Further studies are needed to assess the role played by p62/SQSTM1 and ABCB6 in cellular response to iAs.

From the Nrf2-regulated genes responsive to iAs, *HMOX1*, *SQSTM1*, *GSTM*, *GCLM* and *FTL* showed a dose and time dependent expression, with a clear concordance with the increase in the protein levels of Nrf2. On the other hand, the expression of *GSR*, *MRP2* and *NQO1* were lower and independent of dose and time. In addition, *HMOX1* showed an earlier (20 min) and more sensitive response (0.1 µM) to iAs than *NQO1* exposure. The early differential response in gene expression showed by *HMOX1* and *NQO1* to iAs exposure was also identified at the protein level indicating the biological relevance of this finding. Taken together this new data suggest that transcription factors others than Nrf2 could be important in cellular response to iAs. Since *HMOX1* induction by iAs has been previously detected in different cells types such as macrophages, fibroblasts, keratinocytes and lung epithelial cells [28], [41], [49], this molecule has been proposed as a potentially robust biomarker for iAs exposure. In this study we found that *HMOX1* response in a very fast and sensitive manner to iAs exposure. Therefore expression levels of *HMOX1* in whole blood cells could potentially complement arsenic measurements in urine as a synergistic biomarker of exposure.

The use of a lymphoblastoid immortalized non-transformed cell line allowed us to analyze the cellular responses to iAs without the bias imposed from a tumor derived cell line, where several of the defence mechanisms and cell-cycle may be altered. This model system also benefited us by allowing the capture of the early cellular responses to iAs, which were not previously observed in similar studies performed using lymphoblastoid cells derived from people chronically exposed to iAs [35], [36], [37]. For instance, alterations in the expression of antioxidants and detoxificants genes were not observed in any of these previous studies, but were identified at the doses we used. Likewise, no significant differences in the expression of genes involved in DNA replication were reported. Decrease in the expression of genes involved in immune regulation found in this study has also been reported in previous studies analyzing individuals chronically exposed to iAs [35], [36], [37].

In summary, in this study we found that *HMOX1* transcript showed the highest levels of altered expression and sensitivity in response to acute exposures of iAs at early time points not previously explored in other studies. Protein induction for HMOX1 followed a similar pattern. Although the Nrf2-Keap1 system was the main signalling pathway responsive to iAs exposure in lymphoblastoid cells based on expression levels, additional pathways such as AhR, interferon signalling and glucocorticoid receptor signalling may also play a bigger than expected role in the cellular response to iAs exposure. We also identified novel Nrf2-Keap1 transcripts regulated, such as *SQSTM1* and *ABCB6* in response to acute iAs exposures. Our study also highlights the need for more expansive studies to understand the acute versus chronic exposures involved in iAs metabolism.

## Supporting Information

Figure S1 **Evaluation of the cytotoxic effects of iAs in a lymphoblastoid cell line.** The CL-1 cell line was exposed to 5 µM of iAs for the indicated period times and cell viability evaluated by trypan blue exclusion. Three independent cell cultures were used for each assay data point.
(TIF)

Table S1 **List of differential gene expression in CL-1 cell line expose for 2 hrs of iAs 5 µM.**
(PDF)

Table S2 **List of differential gene expression in CL-1 cell line expose for 12 hrs of iAs 5 µM.**
(PDF)
