# Supplementary figures and images for: The NRF2-KEAP1 Pathway Is an Early Responsive Gene Network in Arsenic Exposed Lymphoblastoid Cells
Source: PLoS One. 2014 Feb 7;9(2):e88069. doi: 10.1371/journal.pone.0088069 (PMC3917856; doi:10.1371/journal.pone.0088069)

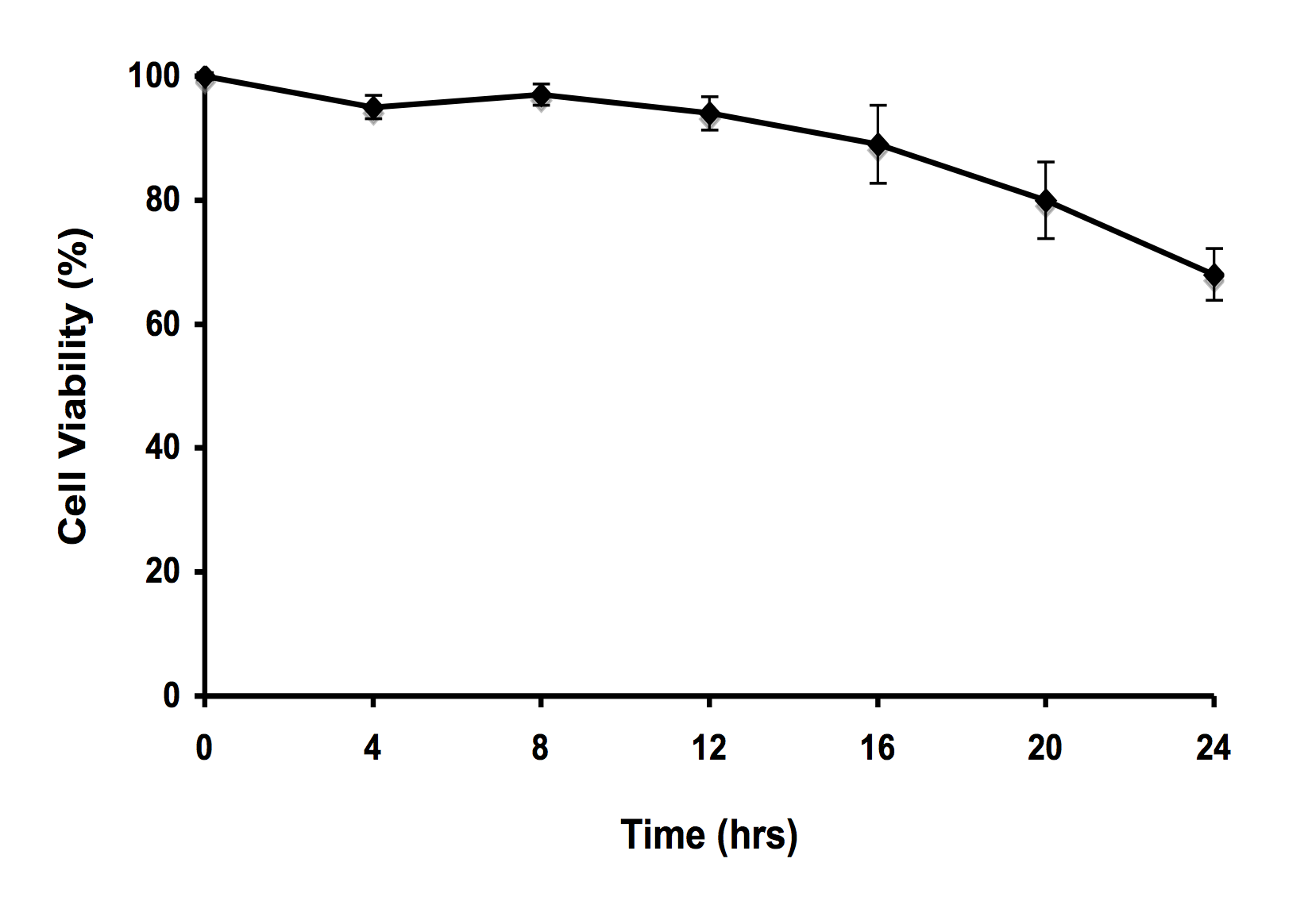

Supplement: Figure S1 — Evaluation of the cytotoxic effects of iAs in a lymphoblastoid cell line. The CL-1 cell line was exposed to 5 µM of iAs for the indicated period times and cell viability evaluated by trypan blue exclusion. Three independent cell cultures were used for each assay data point. (TIF) [file pone.0088069.s001.tif]
