# Supplementary material for: The NRF2-KEAP1 Pathway Is an Early Responsive Gene Network in Arsenic Exposed Lymphoblastoid Cells
Source: PLoS One. 2014 Feb 7;9(2):e88069. doi: 10.1371/journal.pone.0088069 (PMC3917856; doi:10.1371/journal.pone.0088069)
Supplement: Table S1 — List of differential gene expression in CL-1 cell line expose for 2 hrs of iAs 5 µM. (PDF) [file pone.0088069.s002.pdf]

Number	Chromosome	Gene ID	Gene Symbol	Accession Numb	Entrez Gene Na
38	2	10058	ABCB6	AK057026	ATP-binding cas
49	12	217	ALDH2	NM_000690	aldehyde dehyc
54	4	353322	ANKRD37	NM_181726	ankyrin repeat c
61	4	118429	ANTXR2	AK055636	anthrax toxin re
14	10	9531	BAG3	AK291333	BCL2-associate
58	8	665	BNIP3L	NM_004331	BCL2/adenoviru
78	5	4064	CD180	NM_005582	CD180 molecul
74	1	1043	CD52	NM_001803	CD52 molecule
75	17	8851	CDK5R1	NM_003885	cyclin-depender
42	6	1026	CDKN1A	NM_078467	cyclin-depender
23	15	79094	CHAC1	NM_024111	ChaC, cation tra
39	11	26973	CHORDC1	AK290231	cysteine and his
48	1	1185	CLCN6	ENST00000312	The CLCN family o
19	17	1440	CSF3	AK298116	colony stimulati
66	2	58190	CTDSP1	NM_021198	CTD (carboxy-te
25	19	162989	DEDD2	AK130203	death effector d
24	15	55466	DNAJA4	NM_018602	DnaJ (Hsp40) h
6	19	3337	DNAJB1	NM_006145	DnaJ (Hsp40) h
21	1	11080	DNAJB4	AK297985	DnaJ (Hsp40) h
22	7	10049	DNAJB6	AK124827	DnaJ (Hsp40) h
50	6	9750	FAM65B	NM_014722	family with sequ
45	12	2288	FKBP4	AK313918	FK506 binding p
17	11	2495	FTH1	NM_002032	ferritin, heavy p
51	10	170384	FUT11	NM_173540	fucosyltransfera
72	5	2533	FYB	AK297077	FYN binding pro
37	1	2730	GCLM	NM_002061	glutamate-cyste
79	3	2838	GPR15	ENST00000284	G protein-couple
55	5	84868	HAVCR2	AK027334	hepatitis A virus
67	17	3090	HIC1	NM_001098202	hypermethylate
80	6	3007	HIST1H1D	AK312845	histone cluster
1	22	3162	HMOX1	NM_002133	heme oxygenas
53	4	3184	HNRNPD	AK300149	heterogeneous
3	6	3303	HSPA1A	AK303980	heat shock 70kI
2	6	3304	HSPA1B	ENST00000450	This intronless ger
26	4	22824	HSPA4L	AK027822	heat shock 70kI
28	11	3312	HSPA8	AK310467	heat shock 70kI
46	13	10808	HSPH1	ENST00000439123	
77	10	3433	IFIT2	NM_001547	interferon-induc
70	15	3603	IL16	NM_004513	interleukin 16 (ly
43	17	4097	MAFG	AK130699	v-maf musculoa
62	11	219972	MPEG1	ENST00000361050	
7	16	4489	MT1A	NM_005946	metallothionein
18	16	4490	MT1B	NM_005947	metallothionein
16	16	326343	MT1DP	NR_027781	metallothionein
8	16	4493	MT1E	NM_175617	metallothionein
13	16	4494	MT1F	NM_005949	metallothionein
15	16	4495	MT1G	NM_005950	metallothionein
11	16	4496	MT1H	NM_005951	metallothionein

5	16	644314	MT1IP	NR_003669	
9	16	4500	MT1L	NR_001447	metallothionein
33	16	4499	MT1M	NM_176870	metallothionein
4	16	4501	MT1X	NM_005952	metallothionein
12	16	4502	MT2A	NM_005953	metallothionein
56	X	8473	OGT	AK300267	O-linked N-acet
20	5	8974	P4HA2	ENST00000401867	
65	2	5163	PDK1	ENST00000443353	
81	3	5210	PFKFB4	AK312623	6-phosphofructo
52	17	146850	PIK3R6	AK091819	phosphoinositid
76	6	639	PRDM1	NM_001198	PR domain con
71	1	5724	PTAFR	NM_000952	platelet-activatir
57	17	84440	RAB11FIP4	ENST00000394742	
63	X	9363	RAB33A	AK094927	RAB33A, memb
40	11	54734	RAB39	AK292969	RAB39, membe
73	14	6039	RNASE6	AK313580	ribonuclease, R
31	11	871	SERPINH1	AK075504	serpin peptidas
47	2	6574	SLC20A1	AK123420	solute carrier fa
10	1	7779	SLC30A1	NM_021194	solute carrier fa
59	2	57181	SLC39A10	ENST00000430412	
44	11	6520	SLC3A2	NM_001012664	solute carrier fa
41	4	23657	SLC7A11	AK290359	solute carrier fa
34	16	8140	SLC7A5	NM_003486	solute carrier fa
36	6	677806	SNORA20	NR_002960	small nucleolar
27	6	677812	SNORA29	NR_002965	small nucleolar
29	11	85389	SNORD14C	NR_001453	small nucleolar
32	11	85391	SNORD14E	NR_003125	small nucleolar
35	5	26785	SNORD63	NR_002913	small nucleolar
60	4	23158	TBC1D9	NM_015130	TBC1 domain fa
64	4	80008	TMEM156	ENST00000344606	
69	3	8743	TNFSF10	AK296085	tumor necrosis
30	7	90637	ZFAND2A	ENST00000401903	
68	8	55893	ZNF395	NM_018660	zinc finger prote

Log Fold Change
1,149999976
-0,879999995
-0,980000019
-1,070000052
1,909999967
-1,059999943
-1,289999962
-1,230000019
-1,24000001
1,110000014
1,480000019
1,149999976
0,910000026
1,710000038
-1,120000005
1,379999995
1,470000029
2,329999924
1,50999999
1,49000001
-0,910000026
1,059999943
1,870000005
-0,939999998
-1,200000048
1,190000057
-1,299999952
-1,00999999
-1,149999976
-1,360000014
3,130000114
-0,970000029
2,869999886
2,880000114
1,370000005
1,340000033
1,049999952
-1,25
-1,190000057
1,080000043
-1,070000052
2,319999933
1,860000014
1,899999976
2,269999981
1,99000001
1,909999967
2,140000105

2,359999895
2,25
1,24000001
2,779999971
2,049999952
-1,019999981
1,600000024
-1,110000014
-1,419999957
-0,949999988
-1,24000001
-1,190000057
-1,019999981
-1,090000033
1,120000005
-1,220000029
1,25
1,049999952
2,210000038
-1,059999943
1,070000052
1,120000005
1,230000019
1,200000048
1,350000024
1,340000033
1,25
1,210000038
-1,059999943
-1,100000024
-1,159999967
1,269999981
-1,149999976
