# Supplementary material for: The NRF2-KEAP1 Pathway Is an Early Responsive Gene Network in Arsenic Exposed Lymphoblastoid Cells
Source: PLoS One. 2014 Feb 7;9(2):e88069. doi: 10.1371/journal.pone.0088069 (PMC3917856; doi:10.1371/journal.pone.0088069)
Supplement: Table S2 — List of differential gene expression in CL-1 cell line expose for 12 hrs of iAs 5 µM. (PDF) [file pone.0088069.s003.pdf]

| Number | Chromosom | Gene ID | Gene Symbol | Accession Number | Log Fold Change |
|---|---|---|---|---|---|
| 1 | 2 | 10058 | ABCB6 | AK303230 | 1,559999943 |
| 2 | 14 | 63874 | ABHD4 | ENST00000418446 | 1,379999995 |
| 3 | 15 | 23205 | ACSBG1 | AK054882 | -1,299999952 |
| 4 | 4 | 84803 | AGPAT9 | ENST00000264409 | 1,75999999 |
| 5 | 17 | 245 | ALOX12P2 | NR_002710 | 1,289999962 |
| 6 | 9 | 259 | AMBP | AK290837 | 1,75 |
| 7 | 6 | 93663 | ARHGAP18 | NM_033515 | -1,269999981 |
| 8 | 19 | 55723 | ASF1B | AK302134 | -1,210000038 |
| 9 | 4 | 55024 | BANK1 | AK000713 | -1,299999952 |
| 10 | 3 | 151888 | BTLA | NM_001085357 | -1,25 |
| 11 | 6 | 11118 | BTN3A2 | NM_007047 | -1,179999948 |
| 12 | 6 | 10384 | BTN3A3 | NM_197974 | -1,269999981 |
| 13 | 13 | 81617 | CAB39L | NM_030925 | 1,539999962 |
| 19 | 19 | 898 | CCNE1 | NM_001238 | -1,269999981 |
| 20 | 8 | 9134 | CCNE2 | ENST00000396133 | -1,529999971 |
| 21 | 16 | 899 | CCNF | NM_001761 | -1,110000014 |
| 22 | 10 | 983 | CDC2 | AK291939 | -1,320000052 |
| 23 | 22 | 8318 | CDC45L | ENST00000407835 | -1,289999962 |
| 24 | 11 | 113130 | CDCA5 | NM_080668 | -1,379999995 |
| 25 | 2 | 83879 | CDCA7 | NM_145810 | -1,460000038 |
| 26 | 7 | 55536 | CDCA7L | ENST00000405437 | -1,399999976 |
| 27 | 1 | 55143 | CDCA8 | ENST00000373055 | -1,269999981 |
| 28 | 17 | 8851 | CDK5R1 | NM_003885 | -1,370000005 |
| 29 | 2 | 79745 | CLIP4 | AK057267 | 1,519999981 |
| 30 | 14 | 79789 | CLMN | AK022445 | -1,5 |
| 31 | 16 | 1428 | CRYM | AK310424 | -1,450000048 |
| 32 | 22 | 1454 | CSNK1E | NM_001894 | -1,429999948 |
| 33 | 2 | 58190 | CTDSP1 | ENST00000431127 | -1,49000001 |
| 34 | 12 | 10106 | CTDSP2 | AK294930 | -1,159999967 |
| 35 | 20 | 5476 | CTSA | ENST00000354880 | 1,25 |
| 36 | 11 | 1509 | CTSD | AK130178 | 1,24000001 |
| 37 | 4 | 91351 | DDX60L | ENST00000284630 | 1,320000052 |
| 38 | 17 | 79154 | DHRS11 | AK315735 | -1,440000057 |
| 39 | 14 | 9787 | DLGAP5 | NM_014750 | -1,289999962 |
| 40 | 15 | 55466 | DNAJA4 | AK098079 | 1,289999962 |
| 41 | 1 | 11080 | DNAJB4 | AK297985 | 1,629999995 |
| 42 | 7 | 9732 | DOCK4 | ENST00000342288 | 1,25 |
| 43 | 7 | 54749 | EPDR1 | NM_017549 | 1,350000024 |
| 44 | 1 | 9156 | EXO1 | NM_130398 | -1,179999948 |
| 45 | 11 | 374393 | FAM111B | NM_198947 | -1,230000019 |
| 46 | 1 | 54855 | FAM46C | NM_017709 | -1,610000014 |
| 47 | 6 | 9750 | FAM65B | AK299047 | -1,279999971 |
| 48 | X | 55026 | FAM70A | AK289684 | 1,429999948 |
| 49 | 15 | 55215 | FANCI | AK001581 | -1 |
| 50 | 8 | 157574 | FBXO16 | NM_172366 | -1,450000048 |
| 51 | 6 | 26271 | FBXO5 | AK055221 | -1,350000024 |
| 52 | 17 | 2232 | FDXR | AK094120 | -1,090000033 |
| 53 | 11 | 2495 | FTH1 | NM_002032 | 1,730000019 |

| | | | | | |
|---|---|---|---|---|---|
| 54 | 19 | 2512 | FTL | AK026534 | 1,379999995 |
| 55 | 5 | 2533 | FYB | AK297077 | -1,50999999 |
| 56 | 6 | 2729 | GCLC | AK313480 | 1,24000001 |
| 57 | 1 | 2730 | GCLM | NM_002061 | 1,620000005 |
| 58 | 7 | 113263 | GLCCI1 | NM_138426 | -1,220000029 |
| 59 | 19 | 2788 | GNG7 | AK311750 | -1,25 |
| 60 | 3 | 26996 | GPR160 | NM_014373 | -1,179999948 |
| 61 | 12 | 55507 | GPRC5D | NM_018654 | -1,590000033 |
| 62 | 8 | 2936 | GSR | NM_000637 | 1,25 |
| 63 | 5 | 84868 | HAVCR2 | AK027334 | -1,309999943 |
| 64 | 6 | 10866 | HCP5 | AK093953 | -2,089999914 |
| 65 | 4 | 51191 | HERC5 | NM_016323 | 1,320000052 |
| 66 | 4 | 55008 | HERC6 | AK295832 | 1,330000043 |
| 67 | 6 | 3009 | HIST1H1B | NM_005322 | -1,889999986 |
| 68 | 6 | 3006 | HIST1H1C | NM_005319 | -1,340000033 |
| 69 | 6 | 3007 | HIST1H1D | NM_005320 | -1,779999971 |
| 70 | 6 | 3008 | HIST1H1E | NM_005321 | -1,669999957 |
| 71 | 6 | 8335 | HIST1H2AB | NM_003513 | -1,409999967 |
| 72 | 6 | 8334 | HIST1H2AC | NM_003512 | -1,5 |
| 73 | 6 | 3013 | HIST1H2AD | ENST00000341023 | -1,600000024 |
| 74 | 6 | 3012 | HIST1H2AE | AK311824 | -1,769999981 |
| 75 | 6 | 8969 | HIST1H2AG | NM_021064 | -1,50999999 |
| 76 | 6 | 85235 | HIST1H2AH | NM_080596 | -1,620000005 |
| 77 | 6 | 8329 | HIST1H2AI | NM_003509 | -1,429999948 |
| 78 | 6 | 8330 | HIST1H2AK | NM_003510 | -1,370000005 |
| 79 | 6 | 8332 | HIST1H2AL | NM_003511 | -1,50999999 |
| 80 | 6 | 3018 | HIST1H2BB | NM_021062 | -1,75 |
| 81 | 6 | 8347 | HIST1H2BC | ENST00000396984 | -1,659999967 |
| 82 | 6 | 3017 | HIST1H2BD | NM_138720 | -1,669999957 |
| 83 | 6 | 8343 | HIST1H2BF | NM_003522 | -1,330000043 |
| 84 | 6 | 8339 | HIST1H2BG | NM_003518 | -1,840000033 |
| 85 | 6 | 8345 | HIST1H2BH | AK310576 | -1,460000038 |
| 86 | 6 | 8346 | HIST1H2BI | NM_003525 | -1,899999976 |
| 87 | 6 | 8970 | HIST1H2BJ | NM_021058 | -1,600000024 |
| 88 | 6 | 8340 | HIST1H2BL | NM_003519 | -1,669999957 |
| 89 | 6 | 8342 | HIST1H2BM | NM_003521 | -1,860000014 |
| 90 | 6 | 8341 | HIST1H2BN | NM_003520 | -1,25999999 |
| 91 | 6 | 8358 | HIST1H3B | ENST00000244661 | -1,620000005 |
| 92 | 6 | 8352 | HIST1H3C | ENST00000360577 | -1,360000014 |
| 93 | 6 | 8351 | HIST1H3D | NM_003530 | -1,600000024 |
| 94 | 6 | 8968 | HIST1H3F | NM_021018 | -1,289999962 |
| 95 | 6 | 8355 | HIST1H3G | NM_003534 | -1,850000024 |
| 96 | 6 | 8354 | HIST1H3I | ENST00000328488 | -1,830000043 |
| 97 | 6 | 8356 | HIST1H3J | NM_003535 | -1,330000043 |
| 98 | 6 | 8361 | HIST1H4F | NM_003540 | -1,360000014 |
| 99 | 6 | 8294 | HIST1H4I | NM_003495 | -1,409999967 |
| 100 | 6 | 8368 | HIST1H4L | NM_003546 | -1,350000024 |
| 101 | 1 | 317772 | HIST2H2AB | NM_175065 | -1,200000048 |
| 102 | 1 | 337875 | HIST2H2BA | NR_027337 | -1,860000014 |

| | | | | | |
|---|---|---|---|---|---|
| 103 | 1 | 92815 | HIST3H2A | AK311930 | -1,470000029 |
| 104 | 6 | 3112 | HLA-DOB | ENST00000452392 | -1,289999962 |
| 105 | 22 | 3162 | HMOX1 | AK313120 | 4,679999828 |
| 106 | 10 | 3167 | HMX2 | NM_005519 | -1,710000038 |
| 107 | 6 | 3303 | HSPA1A | AK301243 | 2,329999924 |
| 108 | 6 | 3304 | HSPA1B | ENST00000458062 | 2,160000086 |
| 109 | 7 | 3315 | HSPB1 | AK296890 | 1,889999986 |
| 110 | 11 | 10553 | HTATIP2 | NM_006410 | 1,49000001 |
| 111 | 17 | 3384 | ICAM2 | NM_001099786 | -1,620000005 |
| 112 | 10 | 3434 | IFIT1 | NM_001548 | 1,370000005 |
| 113 | 4 | 3512 | IGJ | AK312014 | -1,200000048 |
| 114 | 15 | 3603 | IL16 | NM_004513 | -1,5 |
| 115 | 6 | 3662 | IRF4 | NM_002460 | -1,25 |
| 116 | 16 | 3687 | ITGAX | NM_000887 | -1,399999976 |
| 117 | 10 | 3832 | KIF11 | NM_004523 | -1,149999976 |
| 119 | 3 | 84859 | LRCH3 | ENST00000441090 | -1,149999976 |
| 120 | 2 | 9208 | LRRFIP1 | NM_001137550 | -1,309999943 |
| 121 | 2 | 130576 | LYPD6B | ENST00000409876 | -1,409999967 |
| 122 | 17 | 4097 | MAFG | AK130699 | 1,25999999 |
| 123 | 10 | 55388 | MCM10 | NM_182751 | -1,450000048 |
| 124 | 3 | 4171 | MCM2 | AK128291 | -1,110000014 |
| 125 | 6 | 4172 | MCM3 | ENST00000421471 | -1,320000052 |
| 126 | 8 | 4173 | MCM4 | ENST00000429229 | -1,330000043 |
| 127 | 22 | 4174 | MCM5 | ENST00000444582 | -1,389999986 |
| 128 | 2 | 4175 | MCM6 | AK312575 | -1,159999967 |
| 129 | 7 | 4176 | MCM7 | NM_005916 | -1,720000029 |
| 130 | 6 | 4199 | ME1 | AK302777 | 1,450000048 |
| 131 | 3 | 64747 | MFSD1 | ENST00000361159 | 1,149999976 |
| 132 | 5 | 51237 | MGC29506 | ENST00000417694 | -1,149999976 |
| 133 | 6 | 4276 | MICA | ENST00000455410 | -2,119999886 |
| 134 | 19 | 2872 | MKNK2 | AK293742 | 1,080000043 |
| 135 | 1 | 10962 | MLLT11 | NM_006818 | 1,730000019 |
| 136 | 4 | 22915 | MMRN1 | AK302421 | -1,5 |
| 137 | 4 | 84057 | MND1 | NM_032117 | -1,299999952 |
| 138 | 1 | 1096 | MRP2 | NM_012833 | 1,559999943 |
| 139 | 16 | 4489 | MT1A | ENST00000443255 | 3,359999895 |
| 140 | 16 | 4490 | MT1B | NM_005947 | 3,130000114 |
| 141 | 16 | 326343 | MT1DP | ENST00000262499 | 2,650000095 |
| 142 | 16 | 4493 | MT1E | ENST00000306061 | 3,630000114 |
| 143 | 16 | 4494 | MT1F | NM_005949 | 3,279999971 |
| 144 | 16 | 4495 | MT1G | ENST00000379811 | 3,210000038 |
| 145 | 16 | 4496 | MT1H | NM_005951 | 2,839999914 |
| 146 | 16 | 644314 | MT1IP | NR_003669 | 3,279999971 |
| 147 | 16 | 4500 | MT1L | NR_001447 | 3,119999886 |
| 148 | 16 | 4499 | MT1M | NM_176870 | 2,329999924 |
| 149 | 16 | 4501 | MT1X | NM_005952 | 3,720000029 |
| 150 | 16 | 4502 | MT2A | NM_005953 | 2,99000001 |
| 151 | 8 | 92140 | MTDH | AK000745 | -1,120000005 |
| 152 | 6 | 63915 | MUTED | ENST00000358251 | -1,49000001 |

| | | | | | |
|---|---|---|---|---|---|
| 153 | 6 | 4602 | MYB | NM_001130173 | -1,330000043 |
| 154 | 20 | 4605 | MYBL2 | AK303249 | -1,299999952 |
| 155 | 2 | 4648 | MYO7B | ENST00000272666 | -1,389999986 |
| 156 | 19 | 199713 | NLRP7 | AK302655 | 1,269999981 |
| 157 | 16 | 1728 | NQO1 | NM_001025434 | 1,620000005 |
| 158 | 6 | 4835 | NQO2 | AK311746 | 1,830000043 |
| 159 | 5 | 5019 | OXCT1 | NM_000436 | -1,25999999 |
| 160 | 5 | 8974 | P4HA2 | ENST00000401867 | 1,429999948 |
| 161 | 16 | 5073 | PARN | ENST00000420015 | -1,220000029 |
| 162 | 8 | 55872 | PBK | AK027291 | -1,139999986 |
| 163 | 7 | 5294 | PIK3CG | NM_002649 | -1,340000033 |
| 164 | 17 | 146850 | PIK3R6 | AK091819 | -1,350000024 |
| 165 | X | 8544 | PIR | AK309656 | 1,539999962 |
| 166 | 6 | 135293 | PM20D2 | AK302464 | -1,289999962 |
| 167 | 8 | 5368 | PNOC | NM_006228 | -1,460000038 |
| 168 | 6 | 5696 | PSMB8 | NM_004159 | -1,24000001 |
| 169 | 15 | 5888 | RAD51 | AK313503 | -1,450000048 |
| 170 | 14 | 6039 | RNASE6 | AK313580 | -1,480000019 |
| 171 | 19 | 23521 | RPL13A | AK056837 | -1,25 |
| 172 | 7 | 219285 | SAMD9L | ENST00000437805 | 1,399999976 |
| 173 | 1 | 6402 | SELL | AK225713 | -1,360000014 |
| 174 | 7 | 10371 | SEMA3A | AK289954 | -1,080000043 |
| 175 | 5 | 9037 | SEMA5A | AK307587 | -1,25 |
| 176 | 10 | 118980 | SFXN2 | AK055711 | -1,190000057 |
| 177 | 3 | 55164 | SHQ1 | AK296789 | -1,370000005 |
| 178 | 13 | 10166 | SLC25A15 | ENST00000338625 | -1,149999976 |
| 179 | 17 | 60386 | SLC25A19 | ENST00000375261 | -1,279999971 |
| 180 | 1 | 7779 | SLC30A1 | NM_021194 | 1,610000014 |
| 181 | 12 | 55652 | SLC48A1 | NM_017842 | 2,00999999 |
| 182 | 3 | 6533 | SLC6A6 | ENST00000452151 | 1,519999981 |
| 183 | 4 | 23657 | SLC7A11 | AK290359 | 1,480000019 |
| 184 | 11 | 11309 | SLCO2B1 | NM_007256 | 1,860000014 |
| 185 | 17 | 162394 | SLFN5 | AK303299 | 1,350000024 |
| 186 | 11 | 23642 | SNHG1 | AK095849 | -1,419999957 |
| 187 | 1 | 85028 | SNHG12 | AK092096 | -1,139999986 |
| 188 | 11 | 9304 | SNORD22 | NR_000008 | -1,350000024 |
| 189 | 11 | 9297 | SNORD29 | NR_002559 | -1,50999999 |
| 190 | 11 | 9299 | SNORD30 | NR_002561 | -1,24000001 |
| 191 | 19 | 26816 | SNORD35A | NR_000018 | -1,24000001 |
| 192 | 1 | 692196 | SNORD76 | NR_003942 | -1,330000043 |
| 193 | 5 | 6678 | SPARC | AK299441 | -1,169999957 |
| 194 | 2 | 57405 | SPC25 | NM_020675 | -1,220000029 |
| 195 | 16 | 83985 | SPNS1 | NM_001142448 | 1,389999986 |
| 196 | 5 | 8878 | SQSTM1 | AK025146 | 1,75999999 |
| 197 | 18 | 6760 | SS18 | AK301504 | -1,279999971 |
| 198 | X | 412 | STS | NM_000351 | -1,350000024 |
| 199 | 6 | 117289 | TAGAP | NM_138810 | -1,169999957 |
| 200 | 6 | 6891 | TAP2 | AK299603 | -1,279999971 |
| 201 | 12 | 23329 | TBC1D30 | ENST00000411580 | -1,279999971 |

| | | | | | |
|---|---|---|---|---|---|
| 202 | 6 | 6941 | TCF19 | AK315258 | -1,320000052 |
| 203 | 13 | 7027 | TFDP1 | AK310401 | -1,080000043 |
| 204 | 12 | 79022 | TMEM106C | AK299154 | -1,25 |
| 205 | 11 | 79073 | TMEM109 | AK303961 | -1,340000033 |
| 206 | 19 | 10430 | TMEM147 | NM_032635 | -1,210000038 |
| 207 | 14 | 7127 | TNFAIP2 | NM_006291 | 1,360000014 |
| 208 | 13 | 55504 | TNFRSF19 | AK292874 | -1,25 |
| 209 | 3 | 23043 | TNIK | NM_001161565 | -1,24000001 |
| 210 | 11 | 1200 | TPP1 | NM_000391 | 1,309999943 |
| 211 | 1 | 80342 | TRAF3IP3 | ENST00000456166 | -1,399999976 |
| 212 | 5 | 9319 | TRIP13 | NM_004237 | -1,279999971 |
| 213 | 2 | 7273 | TTN | ENST00000342992 | -1,269999981 |
| 214 | 6 | 81567 | TXNDC5 | NM_030810 | -1,50999999 |
| 215 | 6 | 51465 | UBE2J1 | AK300629 | -1,320000052 |
| 216 | 1 | 23352 | UBR4 | AK025149 | 1,070000052 |
| 217 | 4 | 7345 | UCHL1 | ENST00000381762 | 1,279999971 |
| 218 | 12 | 7374 | UNG | AK291341 | -1,179999948 |
| 219 | 1 | 10451 | VAV3 | AK295166 | -1,529999971 |
| 220 | 15 | 79968 | WDR76 | AK023035 | -1,24000001 |
| 221 | 16 | 197335 | WDR90 | AK093609 | -1,360000014 |
| 222 | 14 | 23503 | ZFYVE26 | ENST00000411699 | 1,100000024 |
| 223 | 8 | 55893 | ZNF395 | ENST00000380254 | -1,450000048 |
| 224 | 10 | 11130 | ZWINT | NM_007057 | -1,110000014 |
